# Which ICD-9-CM codes should be used for bronchiolitis research?

**DOI:** 10.1186/s12874-018-0589-4

**Published:** 2018-11-22

**Authors:** Paul Walsh, Stephen J. Rothenberg

**Affiliations:** 1 Pediatric Emergency Medicine, Sutter Medical Center Sacramento, Sacramento, CA USA; 2 Instituto Nacional de Salud Pública, Centro de Investigación en Salud Poblacional, Cuernavaca, Morelos Mexico

**Keywords:** Bronchiolitis, Wheezing, Asthma, Misclassification, International classification of diseases

## Abstract

**Background:**

Bronchiolitis is a common respiratory disorder in children. Although there are specific ICD-9-CM diagnosis codes for bronchiolitis, the illness is often coded using broader diagnosis codes. This creates the potential for subject misclassification if researchers rely on specific diagnosis codes when assembling retrospective cohorts. Here we challenge the common research practice of relying on specific diagnosis codes for bronchiolitis.

**Methods:**

We examined the use of diagnosis codes for the first episode of bronchiolitis, bronchitis, acute asthma, and bronchospasm and wheezing, in children younger than six and 24 months in the State of California Medic-Aid database. We categorized codes as narrow or broad diagnosis codes. We compared patient, geographic, and temporal characteristics of the different diagnoses codes.

**Results:**

We identified visits from 48,732 children for first episode of wheezing illness. We retained 48,269 who had the diagnosis codes and data of interest. Diagnosis codes for acute asthma were widely used, even in children younger than six months in whom a diagnosis code for bronchiolitis would have been anticipated. The temporal pattern was similar across all diagnoses. Antipyretics were prescribed more often in those with diagnosis codes for bronchiolitis and bronchitis. Other statistically significant differences were too small to usefully distinguish the groups. There was substantial geographic variability in the diagnosis codes selected.

**Conclusion:**

Users of Medic-Aid administrative data should generally favor broad rather than narrow definitions of bronchiolitis and should perform sensitivity analysis comparing broad and narrow definitions.

**Electronic supplementary material:**

The online version of this article (10.1186/s12874-018-0589-4) contains supplementary material, which is available to authorized users.

## Background

Bronchiolitis is one of the most common causes of morbidity in pediatrics [1]. Bronchiolitis is characterized by symptoms of lower airway obstruction, typically characterized by wheezing, chest wall retractions, and later crackles, following a period of coughing and rhinorrhea [2]. Despite its prevalence, bronchiolitis is a diagnosis that invites subject misclassification when using administrative datasets. Bronchiolitis has a variable clinical presentation, competing upper age limits for diagnosis (12 and 24 months), and is characterized by symptoms which have their own International Classification of Diseases Ninth Edition - Clinical Modification ICD-9-CM codes [3, 4]. Even the word ‘bronchiolitis’ when typed into a computer will often autocomplete to ‘bronchitis’ which also has its own ICD-9-CM code and there is little incentive for non-research data entry staff to catch this mistake. The resulting multiplicity of ICD-9-CM codes can betray their own purpose, lead researchers to misclassify patients, and mislead clinicians and policy makers. Here we challenge the common research practice of relying only on narrow diagnosis codes for bronchiolitis.

We examined the diagnosis bronchiolitis in the State of California Department of Health Care Services (DHCS) administrative database. This database captures the state’s Medic-Aid population and is widely used for both research and to inform policy decisions for California and beyond.

Our purpose was to determine whether narrow diagnostic criteria limited to a small number of specific ICD-9-CM diagnosis codes (narrow diagnosis codes) or broader criteria corresponding to a larger number of less specific codes (broad diagnosis codes) should be used by researchers using administrative data.

Our specific aims were to compare the characteristics of samples selected using broad and narrow diagnosis codes for bronchiolitis and estimate the potential for undercounting cases of bronchiolitis if only narrow diagnosis codes are used.

## Methods

### Data source

The State of California Committee for Protection of Human Subjects and the State of California and DHCS approved this study. Data was obtained from the California Medic-Aid Management Information System/Decision Support System (MMIS/DSS). This database includes up to two diagnostic fields per clinical visit, location, and limited demographic information. Data was extracted from the MMIS/DSS, an SQL database, (Microsoft Corporation, Redmond WA) as text files and converted to Stata 14.2 (Statacorp, College Station, TX) for data management and analysis.

We extracted data from 2004 to 2010 inclusive with any of the diagnosis codes in Table 1 in children younger than 24 months chronological age. We assigned incomplete and outdated codes the most specific description attached to them per the most recent ICD-9-CM version in which they were valid. ICD-9-CM codes were checked using Stata’s icd9 suite of commands and www.icd9data.com. (last accessed 8-Aug-2018).

**Table 1:** Summary of ICD 9 codes included in each category

Category	Codes
Bronchiolitis	466, 466.1, 466.11, 466.19
Bronchitis	490, 466.0
Asthma codes (includes reactive airways disease)	493, 493.0, 493.00, 493.01, 493.02, 493.1, 493.10, 493.12, 493.9, 493.90, 493.91, 493.92
Bronchospasm or wheezing	519.11, 786.07

Summary of ICD 9 codes included in each category. The shaded categories were combined to create the narrow diagnosis category and the unshaded combined to create the broad diagnosis category

We created two categories, narrowly and broadly coded bronchiolitis. The former group included the ICD-9-CM diagnosis codes for bronchiolitis and bronchitis, the latter group included, in addition to the narrow diagnosis group diagnosis codes for bronchospasm, acute asthma, and wheezing. When both a broad and narrow diagnosis code was listed for the same patient visit we coded the visit as having a narrow diagnosis code. We expect that bronchiolitis in younger children would be diagnosed more confidently than in older children, resulting in more specific diagnostic codes. At 24 months of age, the diagnosis bronchiolitis for a first episode should be very common and a diagnosis of asthma very uncommon. This effect should be even more pronounced in children younger than 12 months, and in those children younger than six months of age a diagnosis of asthma should be exceedingly rare. This is because asthma, by definition, requires recurrent episodes and therefore should not be diagnosed at a first episode of wheezing [5]. Age limits vary by source but 12 and 24 months are commonly applied [6–8]. Bronchitis appears to sometimes used almost synonymously with bronchiolitis in some primary care settings [9].

### Comparison of samples generated using narrow and broad diagnosis codes

#### Samples size

We compared the size of samples that would result from using broad and narrow diagnostic criteria for bronchiolitis.

#### Year, season, and month

We compared diagnosis category used by year using percentages. Our large sample size would allow unimportant differences to reach statistical testing and, so we did not perform significance testing. We graphically compared diagnosis category by month of year. We included whether the episode occurred during bronchiolitis season (as retrospectively defined by the CDC) as a variable in a hierarchical multinomial logistic regression with children nested in counties.

#### Child level variables

We identified children younger than 24, 12, and six months of age who presented with a first episode consistent with bronchiolitis. We compared the use of narrow and broad diagnosis codes across each age group. We compared the use of narrow diagnosis versus asthma and bronchospasm codes (i.e. broad diagnostic codes excluding narrow diagnostic codes) across child level variables, age, gender, and prescription of antipyretics using hierarchical Poisson regression with children nested in counties. We used prescription of antipyretics as a surrogate marker for fever because our administrative dataset did not contain any clinical examination data. We used hierarchical multinomial logistic regression with children nested in counties to compare each of the four groups of diagnosis codes comprising the narrow and broad diagnosis codes.

We compared incidence rate ratios between the diagnosis groups shown in Table 1 for subsequent visits for recurrent wheezing within one year of the index visit using hierarchical Poisson regression with children nested in counties adjusted for age, gender, antipyretic prescription, and whether the first presentation occurred during bronchiolitis season. We also performed survival analysis (with censoring at last known visit if follow up was incomplete) and plotted Kaplan Meier failure curves for time to first doctor visit for subsequent wheezing illness and for time to any subsequent visit (allowing for multiple subsequent visits). We adjusted these curves for whether enrolment occurred during bronchiolitis season (code is in Additional file 1).

#### County differences

We compared the odds ratio of a child being assigned a narrow rather than a broad diagnosis code using logistic regression. We used Los Angeles County as the referent county because more children were seen there than in any other county. This informed our decision to use hierarchical modeling with children nested in counties when examining child characteristics. We used choropleths (2010 county boundaries and associated datasets are in Additional file 2, Additional file 3 and Additional file 4) to show the proportion of narrow to broad diagnosis codes used by county for children younger than 6 and 24 months [10].

#### Comparison with published descriptions

We compared the characteristics of narrowly and broadly defined bronchiolitis with a convenience sample of published descriptions. We did not attempt any formal meta-analysis of published descriptions, rather those we present provide a range of published estimates to allow the reader judge whether a cohort of bronchiolitis constructed using narrow or broad criteria better fits typical descriptions of bronchiolitis. We compared age, gender, and used antipyretic prescription as a proxy for fever. We included only non-overlapping publications that did not rely on ICD coding for subject inclusion. Different publications were included as needed to obtain variables of interest (e.g. number with fever rather than mean temperature).

## Results

We identified visits from 48,732 children for first episode of wheezing illness. We retained 48,269 who had the diagnosis codes of interest. Broad criteria (e.g. asthma, bronchospasm) were widely used, even when based on the age less than six months and first episode of wheezing a narrow diagnostic code would have been anticipated. Some cases, 1432/48,732 (3%), were coded using the ICD-9-CM code 466.1 despite this code ceasing to be valid since September 1996. A very small number of cases, 148/48,732 (0.3%), were coded as 466, an incomplete (acute bronchitis/bronchiolitis) and therefore invalid code that was nonetheless present in the state database. These findings reflect the underlying data generating process which relied heavily on numerous human coders using different coding manuals, different software in different locations.

### Samples sizes using broad and narrow classification

Using broad rather than narrow diagnosis codes approximately doubles the number of included children across all age groups. (Table 2).

**Table 2:** Frequency of different diagnosis codes

	Aged ≤6 months	Aged ≤12 months	Aged ≤24 months
Broad Diagnosis	18, 773 (100)	35, 645 (100)	48, 269 (100)
Bronchospasm codes	556 (3.0)	971 (2.7)	1291 (2.7)
Asthma codes	8714 (46.4)	17, 200 (48.3)	24, 197 (50.1)
Bronchiolitis codes	5420 (28.9)	9367 (26.3)	11, 367 (23.6)
Bronchitis codes	4083 (21.8)	8107 (22.7)	11, 414 (23.6)
Narrow Diagnosis	9503 (50.6)	17, 474 (49.0)	22, 781 (47.2)
Bronchiolitis codes	5420 (28.9)	9367 (26.3)	11, 367 (23.6)
Bronchitis codes	4083 (21.8)	8107 (22.7)	11, 414 (23.6)

Frequency of different diagnosis codes and categories for each age group. The broad diagnosis category includes all children. The narrow diagnosis classification includes bronchiolitis and bronchitis codes. The broad classification includes the narrow diagnosis codes plus codes for bronchospasm and asthma. Percentages are in parentheses and may not sum to 100 due to rounding

### Child level variables

We found statistically significant differences in some child characteristics between those with narrow and broad diagnosis codes. These changes persisted after adjustment for county of residence. Although statistically significant, almost none of these effects were large enough to usefully distinguish between the assignment of a broad and narrow criteria. These are shown in Table 3**.**

**Table 3:** Child level variables and diagnosis code used

	Age ≤ 24 months			Age ≤ 12 months		
	IRR	95% lb	95% ub	IRR	95% lb	95% ub
Narrow diagnosis	1	–	–	1	–	–
Asthma and bronchospasm codes						
Age (months)	0.99	0.99	1.00	0.99	0.98	1.00
Age ≤ 6 months	1.04	1.00	1.08	1.01	0.96	1.07
Antipyretic prescribed	7.36	7.02	7.73	7.16	6.79	7.55
Male gender	0.97	0.95	1.00	0.98	0.95	1.01
Bronchiolitis season	1.08	1.05	1.11	1.09	1.05	1.12
Component categories	Age ≤ 24 months			Age ≤ 12 months		
	OR	95% lb	95% ub	OR	95% lb	95% ub
Bronchiolitis codes	1	–	–	1	–	–
Bronchitis codes						
Age (months)	1.07	1.06	1.07	1.07	1.07	1.09
Age ≤ 6 months	0.87	0.80	0.95	0.88	0.88	0.99
Antipyretic prescribed	0.75	0.68	0.83	0.74	0.74	0.82
Male gender	0.94	0.89	1.00	0.94	0.94	1.01
Bronchiolitis season	0.62	0.58	0.65	0.63	0.63	0.67
Asthma codes						
Age (months)	1.07	1.06	1.08	1.09	1.09	1.09
Age ≤ 6 months	0.80	0.73	0.88	0.88	0.88	0.88
Antipyretic prescribed	0.02	0.02	0.02	0.02	0.02	0.02
Male gender	1.09	1.02	1.15	1.08	1.08	1.08
Bronchiolitis season	0.56	0.53	0.60	0.56	0.56	0.56
Bronchospasm codes						
Age (months)	1.05	1.03	1.07	1.02	1.02	1.02
Age ≤ 6 months	0.93	0.75	1.15	0.87	0.87	0.87
Antipyretic prescribed	0.03	0.03	0.04	0.03	0.03	0.03
Male gender	1.03	0.89	1.18	1.03	1.03	1.03
Bronchiolitis season	0.69	0.60	0.79	0.67	0.67	0.67

Top panel: Hierarchical Poisson regression for narrow (referent) versus asthma and bronchospasm codes (i.e. broad diagnosis codes excluding narrow diagnosis codes) Bottom panels: Hierarchical multinomial logistic regression for each component of theses (bronchiolitis codes referent). *IRR*; incident rate ratio, *OR*; odds ratio, lb.; 95% confidence interval lower bound, 95% ub; 95% confidence interval upper bound

**Fig. 1:**
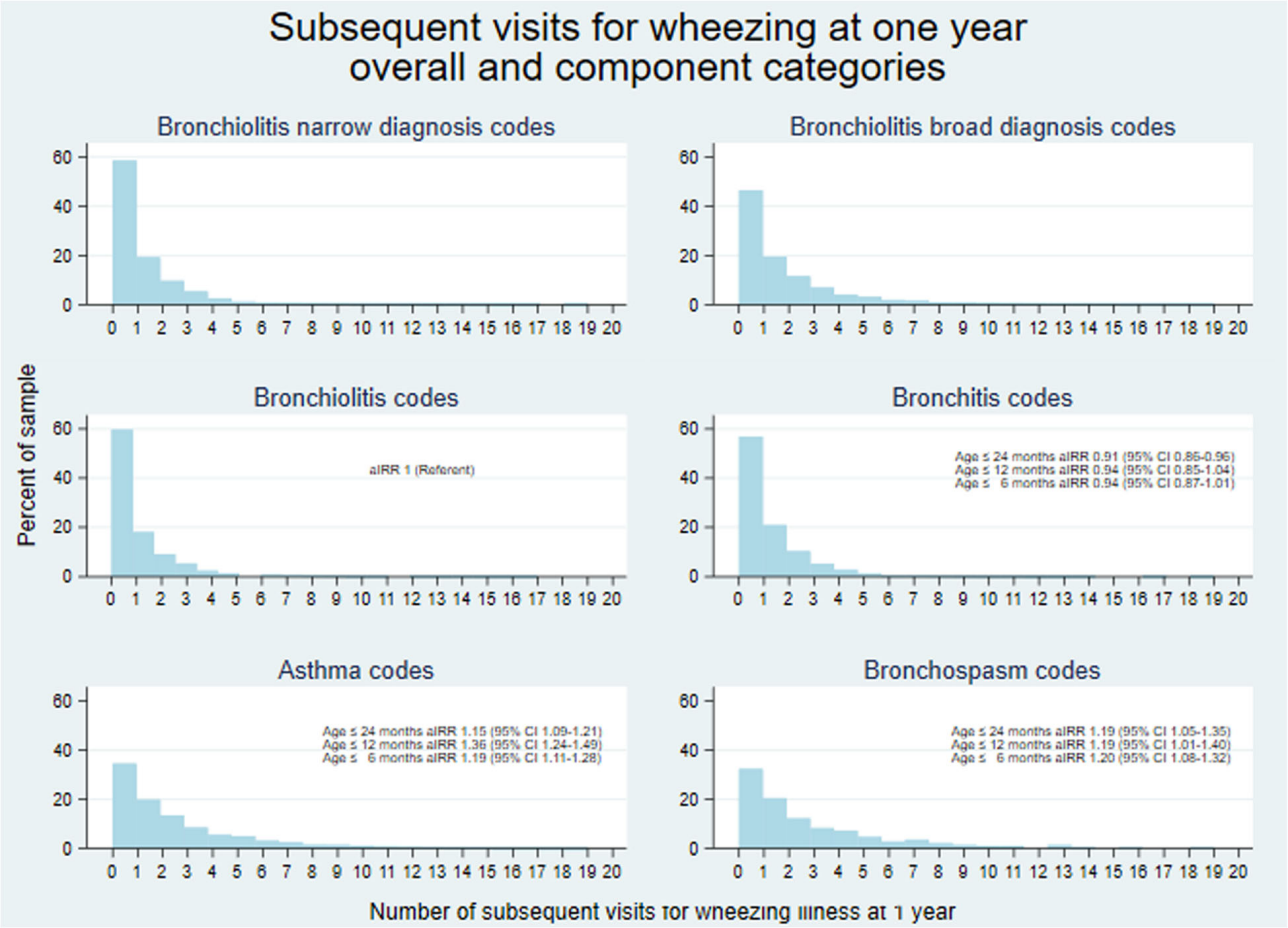
Histogram of subsequent visits for wheezing within 12 months of first wheezing illness by initial diagnosis codes

**Fig. 2:**
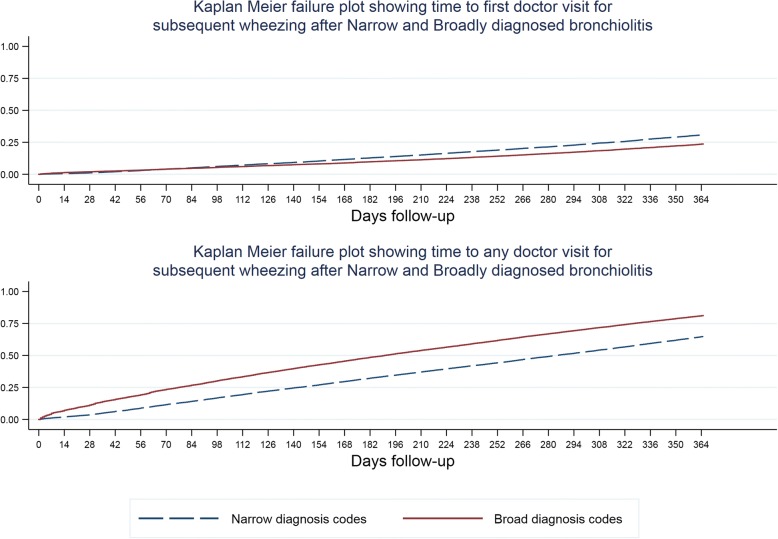
Time to first subsequent visit and any subsequent visit following a first visit for an illness which was coded with narrow or broad codes consistent with bronchiolitis. Broad diagnosis codes encompass narrow ones so statistical tests comparing the two categories were not performed

We also noted increased incidence rate ratio for subsequent visits for wheezing episodes at one year follow up for those with diagnosis codes for asthma or bronchospasm diagnosis codes compared with those with narrow diagnosis codes. The broad diagnosis category includes all children. (Figure 1**,** code in Additional file 5.) The Kaplan Meier curves for time to first subsequent doctor visit for wheezing and any subsequent visit are shown in Fig. 2**.**

### County differences

**Fig. 3:**
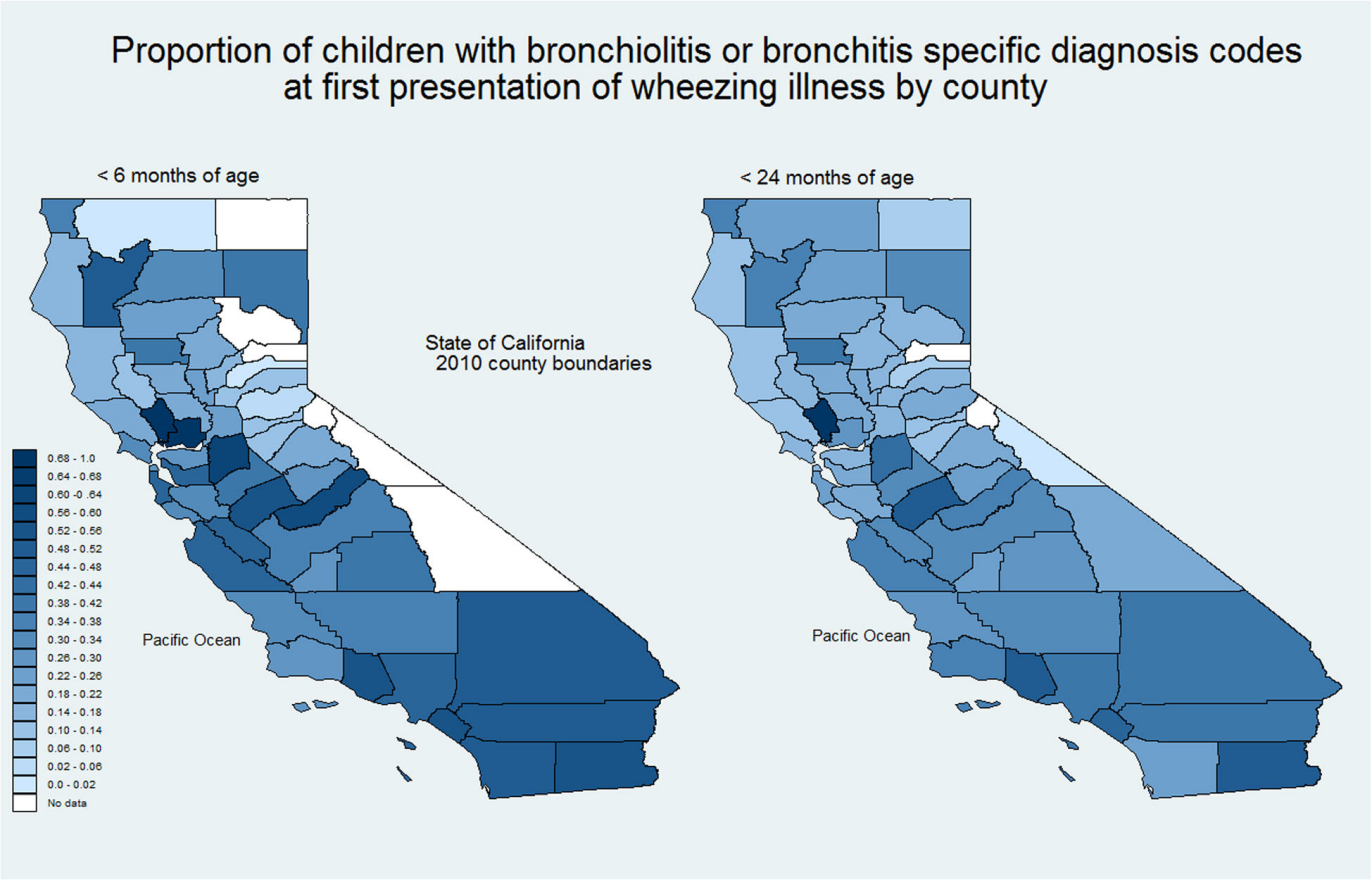
Choropleth showing the proportion of children applied with a narrow rather than broad diagnosis code for bronchiolitis by county using 2010 county boundaries for California

**Fig. 4:**
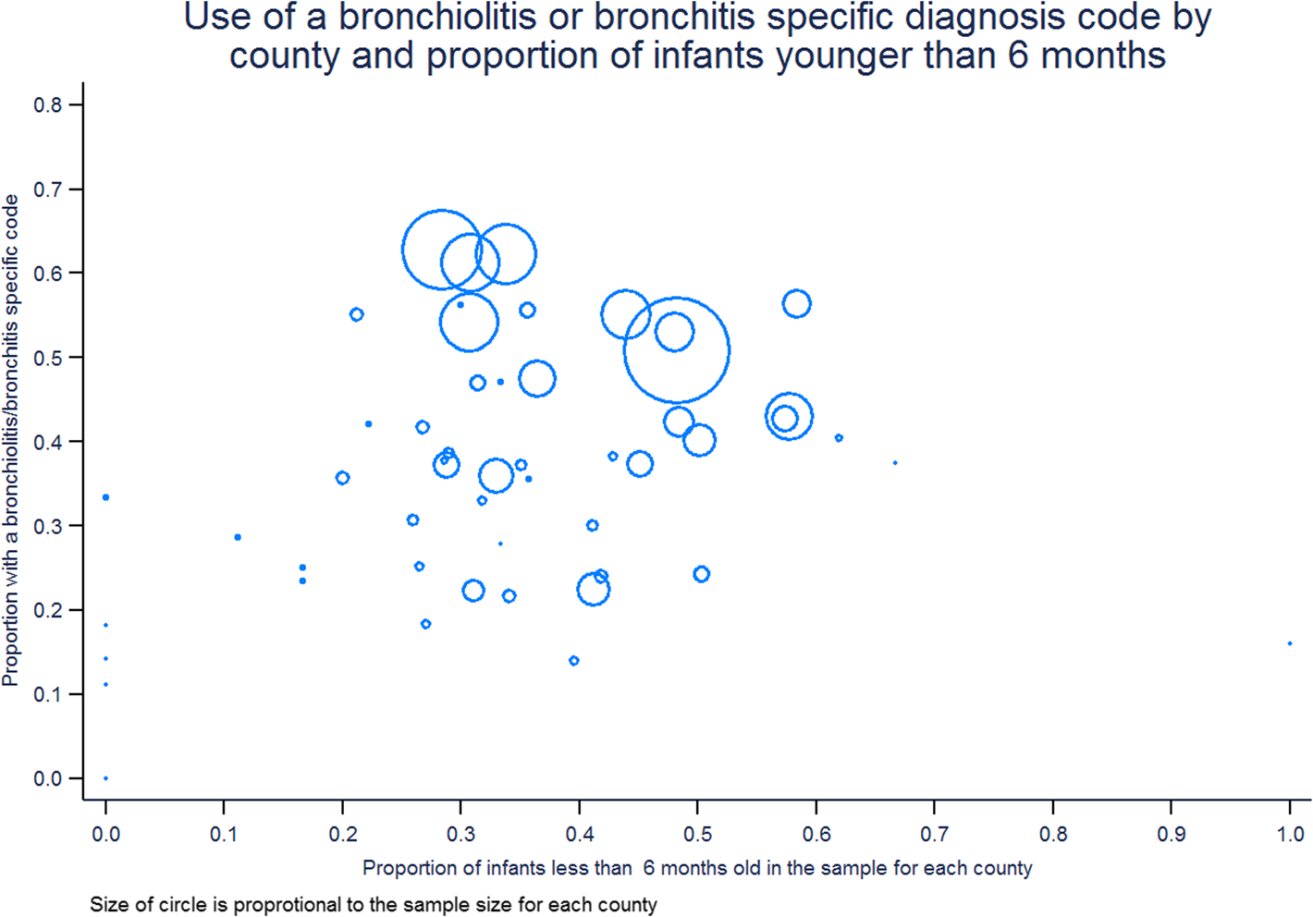
Scatterplot showing the proportion of children assigned a narrow diagnosis code to the proportion of children younger than six months seen for a first wheezing episode for each county

Taking Los Angeles County as referent, the odds of narrow diagnosis code for bronchiolitis being assigned ranged from 0.08 (95% CI 0.02, 0.34) for Amador County to 1.63 (95% CI 1.52, 1.74) for Ventura County. Figure 3 is a choropleth showing the proportion (darker means higher proportion) of children younger than six and 24 months with a narrow diagnosis codes for bronchiolitis in each county. Figure 4 shows the proportion of children assigned a narrow diagnosis code by proportion of the sample younger than 6 months of age for each county. One would expect a broad increase in the use of narrower diagnosis codes in those counties where more children were younger at first presentation, but we did not observe this.

### Year, season, and month

**Fig. 5:**
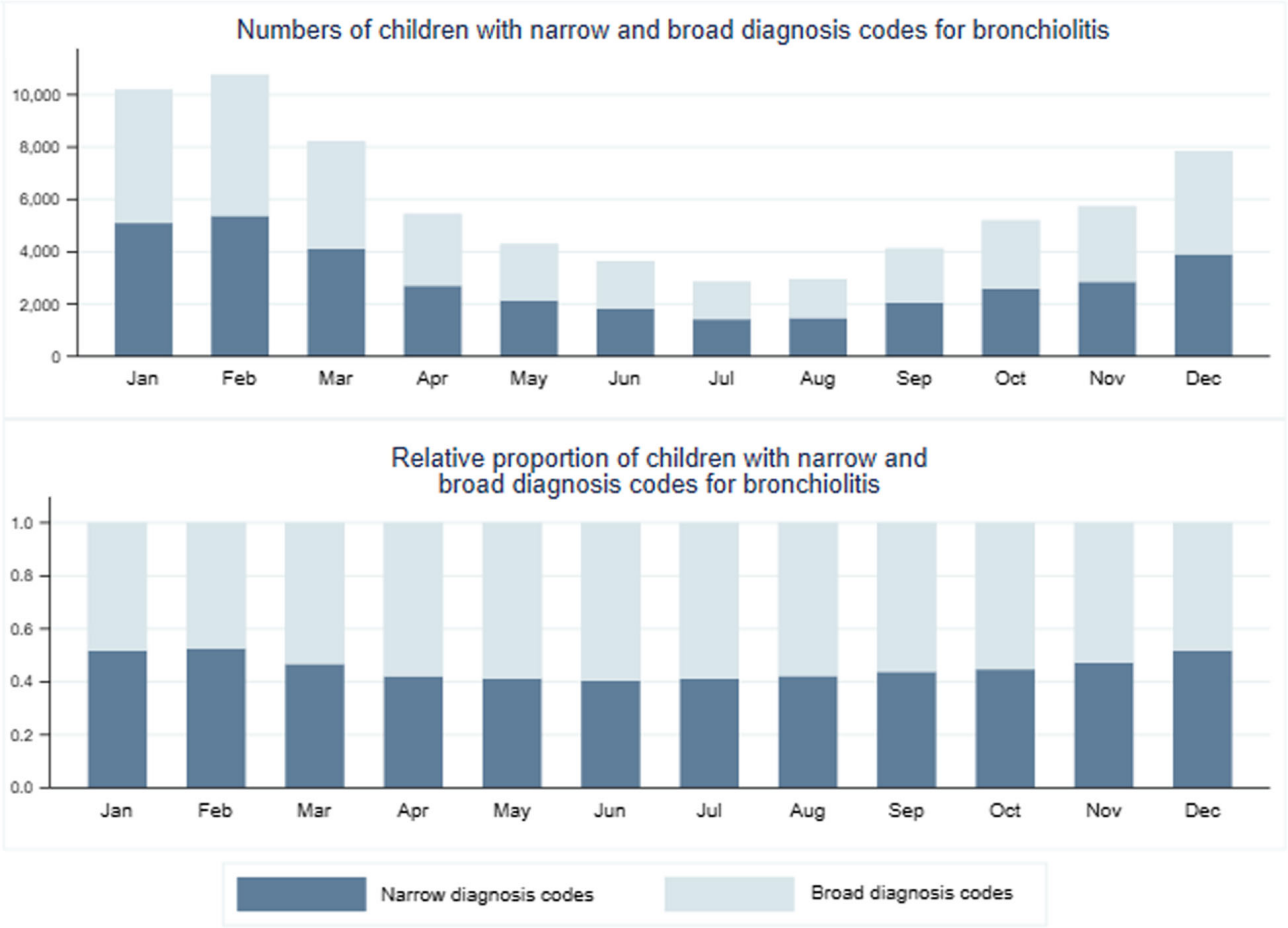
Bar graph of actual and relative frequency of narrow and broad bronchiolitis diagnosis codes in children up to 24 months of age with first episode of wheezing by month of year

Narrow rather than broad diagnostic codes were selected between 49 and 54% of first visits depending on the year with no secular trend noted. Bronchiolitis season typically includes winter and spring, and is retrospectively defined for each year by the Centers for Disease Control and Prevention (CDC), was a significant predictor of the assignment of a narrow diagnosis code. However, when viewed graphically by month of diagnosis, similarity rather than difference is observed. (Figure 5).

### Comparison with published descriptions

Table 4 shows comparisons between published descriptions of bronchiolitis and that which researchers would note if they used narrow or broad definitions of bronchiolitis. In each variable examined, broad rather than narrow diagnosis codes led to a sample more closely matching published descriptions of bronchiolitis. The largest difference was in the prevalence of fever compared with our surrogate marker of antipyretic prescription.

**Table 4:** Comparison of findings in between broad and narrow categories with comparison studies

	Diagnosis	Subject source	Age limit (months)	Study numerator	Study denominator	%
Fever	Broad	All	24	25,514	48,269	52.9
	Broad	All	12	19,107	35,645	53.6
	Narrow	All	24	20,439	22,781	89.7
	Narrow	All	12	15,638	17,474	89.5
	Walsh 2004 [11]	Inpatient	24	28	118	23.7
	Chee 2010 [12]	ED	18	206	640	32.6
	Plint 2004 [8]	ED	12	75	237	31.6
	Ecochard-Dugelay [13]	ED	24	448	821	54.5
Gender	Broad	All	24	26,638	48,269	55.2
	Broad	All	12	19,713	35,645	55.3
	Narrow	All	24	11,909	22,781	52.3
	Narrow	All	12	9189	17,474	52.3
	Walsh 2004 [11]	Inpatient	24	70	118	59.1
	Chee 2010 [12]	ED	18	371	640	57.97
	Corneli [14]	ED	12	368	598	61.5
	Plint [8]	ED	12	140	237	59.1
Age				Study N	Mean	Median
	Broad	All	24	48,269	8.7	7.4
	Broad	All	12	35,645	5.8	6.0
	Narrow	All	24	22,781	8.3	7.0
	Narrow	All	12	17,474	5.9	5.6
	Walsh 2004 [11]	Inpatient	24	118	6.0	
	Chee 2010 [12]	ED	18	640	6.4	4.8
	Corneli [14]	ED	12	598		5.1
	Plint 2004 [8]	ED	12	237		5.0
	Plint 2009 [15]	ED	12	800		5.0

Comparison of findings in between broad and narrow diagnosis codes with comparison studies which did not rely on ICD coding for subject inclusion. *ED*; emergency department

## Discussion

We found that most children who probably had bronchiolitis were instead assigned other ICD-9-CM codes likely reflecting extensive diagnosis misclassification. This variability in how first episodes of wheezing in very young children (which will overwhelmingly be bronchiolitis) is not accounted for by recognizable disease or child characteristics contained in administrative databases. This could mean that clinicians are distinguishing distinct disease processes. Our other findings, and the requirement for a diagnosis of asthma that children be older and the wheezing be recurrent, argue against such an interpretation. Consequently, researchers or health system planners who rely on only on narrow diagnosis codes will miss children who should be included in analyses. The numbers missed would be large – a cohort based on broad diagnosis codes will likely be twice the size of one relying on only narrow codes.

In general, those children diagnosed with narrow diagnosis codes were similar to those with broad ones. A point of distinction between narrowly and broadly classified bronchiolitis was the increased prescription of antipyretics in those with narrow diagnosis codes. This contrasts with operationalized definitions of bronchiolitis which do not include fever, and with clinical descriptions of bronchiolitis which note the presence of fever in 32 to 55% [3, 12, 13, 16]. This compares with our observed prevalence of antipyretic prescription in 90% of narrowly and 53% in broadly classified bronchiolitis. Using the broad diagnosis codes better replicates the sample that researchers and healthcare planners would obtain if they did a prospective study. Other individual level differences, although statistically significant given our sample size, were not of sufficient magnitude to usefully distinguish the narrowly and broadly classified groups.

We also noted a wide geographic variation in diagnostic labels applied. It is unclear why this should be the case given the similarities in the other characteristics of the children.

Much of what we observed represents the misuse of ‘asthma’ as a diagnosis since these codes were heavily used in first-time presentations in children younger than six months. We speculate that there may be a tendency to simplify wheezing diagnoses to ‘asthma’, particularly in busy high-volume clinics where poor health literacy, language barriers, and poor per-patient reimbursement conspire against providers having the time to explain the differences between asthma and bronchiolitis. There may be price to be paid; parents who can be taught to anticipate and tolerate refractory wheezing in bronchiolitis may expect asthma to resolve quickly, and reattend when it does not.

Some of our findings may reflect genuinely competing diagnostic beliefs. The natural history of bronchiolitis is period of upper respiratory tract findings, followed as the virus moves down the respiratory tract by the development of wheezing as the bronchioles become infected, to diffuse crackles as the alveoli become involved. By coding ‘wheezing ‘, ‘asthma’, or ‘bronchitis’ the clinician sidesteps the diagnosis of bronchiolitis for another day. Currently, such misclassification is mostly harmless at the individual patient level. However, as antivirals for common causes of bronchiolitis (e.g. respiratory syncytial virus) become available diagnostic delay will preclude the child from receiving treatment which must be started early to be effective.

Some of our findings may arise from poor coding by non-clinicians (e.g. bronchitis for bronchiolitis) since these data precede widespread physician entry of ICD codes. We speculate that where non-clinically trained staff performed manual coding the presence of a fever may have led to a preference for diagnoses ending in ‘itis’. The authors have personally witnessed data entry clerks shorten bronchiolitis to bronchitis. Moreover, even when diligent, coders may be forced into asthma codes where a clinician diagnosed ‘reactive airways disease’ in the 12 to 24-month period of a child’s life.

Clinicians are also imprecise. Perhaps some clinicians are more willing to diagnose bronchiolitis or bronchitis in the presence of a fever. Interrater agreement for auscultatory findings in children is slight [17]. We have observed providers using stethoscopes that are too large to allow accurate description of adventitial breath sounds in children, and have even overheard the sentiment that diagnostic precision is unimportant. Even without competing diagnostic beliefs, the scene has been set for misclassification of bronchiolitis.

In short, there appears to be a mixture of coding imprecision, diagnostic imprecision, and diagnostic substitution at play. The overuse of asthma as a diagnosis has been commented on elsewhere with a plea by some to relegate ‘asthma’ to a symptom rather than a diagnosis [18]. Our data support this sentiment.

The implications for researchers and consumers of research that relies on State of California DHCS datasets is that careful thought must be given to how cohorts of bronchiolitis are assembled. These data lead us to recommend that while researchers should generally favor a broad definition, they should also use a narrow definition of bronchiolitis and perform sensitivity testing. The narrow definition should typically include the codes 466, 466.0, 466.1, 466.11, 466.19, and 490 with an age limit. The broad definition should add the acute codes for 493, 493.0, 493.00, 493.01, 493.02, 493.1, 493.10, 493.12, 493.9, 493.90, 493.91, and 493.92 and the codes 519.11, 786.07 for bronchospasm and wheezing again with an age limit. Codes should not be excluded for being ‘out of date’ as coders may have used older manuals or software.

Since the underlying data generating mechanism for California Medic-Aid data is similar to Medic-Aid programs in the other 49 states, our findings are similarly broadly generalizable. These findings likely also generalize to other administrative databases used in health services research. California Medic-Aid data is considered of high quality and comparable to that of other large states [19].

Our specific recommendations will diminish in importance as ICD-10 replaces ICD-9-CM but will remain relevant for longitudinal and other research which relies on ICD-9-CM codes for long term or older cohorts. Simply mapping ICD-9-CM codes to ICD-10 will mask rather than quantify the problem. The general lessons of our work however only increase as ICD-10 and ICD-11 are implemented. This is because the proliferation of new codes in ICD-10 increases the potential for missing cases if only narrow diagnosis codes are used for case finding.

Current controversial guidelines advising against the use of bronchodilators in bronchiolitis will inevitably lead to diagnostic substitution as physicians strive to avoid censure by non-medical chart reviewers while still offering their patients a sometimes-effective treatment [20–22].

Our findings may also generalize to other broadly defined diagnoses in which multiple codes could be applied; this problem is likely to increase with the introduction of ICD-10. Others have found variable sensitivity and specificity when relying on diagnosis codes in administrative data sets in adult pulmonary and cardiovascular medicine [23, 24]. Although not directly comparable to our work, their results parallel ours.

### Limitations

Limitations inherent to this work include its retrospective nature, the lack of clinical data points within the MMIS/DSS database itself, the availability of only two diagnostic fields per child, reliance on a single source of data. Consequently, there is no way to independently verify the accuracy of the clinical observations that led to the diagnosis.

Our work relies on the widely accepted assumptions that asthma should generally not be diagnosed in first-time wheezing before 12 or 24 months of age. Our grouping of age and diagnosis categories is reasonable but alternative groupings could have been constructed potentially leading to different results. Achieving substantially different results would require very arbitrary changes. We included 12 and 24-month age cut-offs to address differing viewpoints as to what the upper age limit should be for the diagnosis of bronchiolitis. We included a six-month upper age limit to demonstrate the stability of our results at an age where there is no debate as to whether first time lower airway obstruction or wheezing should be classified as bronchiolitis rather than asthma.

We were unable to address those rare cases of new onset congestive heart failure presenting as or in conjunction with bronchiolitis if a cardiac diagnosis was not specified. However, our aim here is to show the potential for misclassification of groups rather than individuals. We could not determine if death rates differed between broad and narrow diagnosis codes, although death would be rare in this age group.

We compared documented fever in comparison studies with filled antipyretic prescription here. This is a surrogate marker which may tend to overestimate fever as providers could give parents a prescription in anticipation of a fever that may not materialize.

Other limitations include the incomplete follow-up inherent in Medic-Aid populations. Annual Medic-Aid renewal was not automatic in California during the study period. In the event of an emergency department visit or hospitalization parents could retroactively re-enroll (and would be facilitated by hospital billing departments in doing so); so for some parents the difficulties of re-enrolling themselves may have outweighed the perceived benefits [25]. We have carried out this work in a single state, and results may differ if this study was replicated in other states.

Finally, absent real-time observation of clinical encounters, there can be no gold standard to determine which codes most faithfully reflect bronchiolitis. This is an inherent limitation in health services research.

## Conclusions

Users of administrative data should generally favor age-limited broad rather than narrow definitions of bronchiolitis and should perform sensitivity analysis comparing broad and narrow definitions prior to relying only on narrow definitions.

## Additional files

Additional file 1: Map_data.dta. Stata data file. (DTA 26 kb)

Additional file 2: County_map. Stata do file –cleaning. (PDF 25 kb)

Additional file 3: County_map.do. Stata do file –plotting. (DO 1 kb)

Additional file 4: BMC_histogram_combined_fig. Stata do file. (PDF 25 kb)

Additional file 5: Stata_do_file_survival_graph. Stata do file. (PDF 26 kb)
