# Supplementary material for: Which ICD-9-CM codes should be used for bronchiolitis research?
Source: BMC Med Res Methodol. 2018 Nov 22;18:149. doi: 10.1186/s12874-018-0589-4 (PMC6249877; doi:10.1186/s12874-018-0589-4)
Supplement: Supplementary file 2 — County_map. Stata do file –cleaning. (PDF 25 kb) [file 12874_2018_589_MOESM2_ESM.pdf]

```
//
//

//
//
clear
use "C:\Users\8core\Documents\Bronchiolitis_ibuprofen\Summary for Steve\QE B\data for QE B.dta"

//Skip as already done and time consuming

forval x = 1(1)40 {

bys id (dos) : replace county =county[`x'] if county ==""

}


    //County FIPS codes
gen matchvar =trim(county)
merge m:1 matchvar  using "C:\Users\8core\Documents\Bronchiolitis_ibuprofen\Maps\county_fips.dta", gen(_cty_merge)
replace CountyFIPSCode ="75" if county =="San Francisco"
replace GUName = "San Francisco" if regexm(county,"San Francisco")    //missing county details

cap drop cty_visits
  bys GUName  : gen cty_visits = _N
   lab var cty_visits "Number of vists per county"


cap drop cty_br_clean
cap drop cty_br_noisy
cap drop cty_br_ratio
cap drop cty_br_any
cap drop cty_br_clean_prop


bys GUName :egen cty_br_clean = total(bronchiolitis_clean)
bys GUName :egen cty_br_noisy = total(bronchiolitis_noisy)
bys GUName :egen cty_br_any = total(bronchiolitis_any)
bys GUName : gen cty_br_ratio = cty_br_clean / cty_br_noisy
    replace cty_br_ratio=0 if cty_br_ratio ==.

bys GUName :gen cty_br_cl_prop = cty_br_clean/cty_br_any

//County totals

drop if county =="" |county =="UNKNOWN"
 cap rename CountyFIPSCode  fips
 cap drop county_code
 replace fips = "075" if  GUName =="San Francisco"

 gen long fips1 = real(fips)


 stop
#delimit ;


collapse agelt6 age male2
    cty_br_clean cty_br_noisy cty_br_ratio cty_br_cl_prop  fips1 bronchiolitis_clean bronchiolitis_noisy bronchiolitis_an
    (count) cty_visits fips1

    ,by(GUName)
;
 #delimit cr


```

```
save "C:\Users\8core\Documents\Bronchiolitis_ibuprofen\Maps\test1.dta" ,replace

clear
use cadb.dta

merge 1:1  GUName using "C:\Users\8core\Documents\Bronchiolitis_ibuprofen\Maps\test1.dta"


spmap  cty_br_cl_prop using cacoord.dta  ,  id(id) fcolor(Greens2) clnumber(8)


```
