# Supplementary material for: Which ICD-9-CM codes should be used for bronchiolitis research?
Source: BMC Med Res Methodol. 2018 Nov 22;18:149. doi: 10.1186/s12874-018-0589-4 (PMC6249877; doi:10.1186/s12874-018-0589-4)
Supplement: Supplementary file 4 — BMC_histogram_combined_fig. Stata do file. (PDF 25 kb) [file 12874_2018_589_MOESM4_ESM.pdf]

```
///
///Revisions for BMC
//
//


// use "C:\Users\8core\Documents\Bronchiolitis_ibuprofen\Maps\CA_counties\QE_B_for_maps_adjusted.dta"


  hist d365 if d365 <20 & cat ==1 , percent xlab(0/20) bin(20) ylab(0(20)60) ylabel(,angle(0)) title(Bronchiolitis codes)
  gr play fig_hist_indiv.grec
   graph save Graph "C:\Users\8core\Documents\Bronchiolitis_ibuprofen\Maps\BMC revision\graph_cat1.gph" ,replace

    hist d365 if d365 <20 & cat ==2 , percent xlab(0/20) bin(20) ylab(0(20)60) ylabel(,angle(0)) title(Bronchitis codes)
      gr play fig_hist_indiv.grec
       graph save Graph "C:\Users\8core\Documents\Bronchiolitis_ibuprofen\Maps\BMC revision\graph_cat2.gph",replace


  hist d365 if d365 <20 & cat ==3 , percent xlab(0/20) bin(20) ylab(0(20)60) ylabel(,angle(0)) title(Acute asthma codes)
   gr play fig_hist_indiv.grec
    graph save Graph "C:\Users\8core\Documents\Bronchiolitis_ibuprofen\Maps\BMC revision\graph_cat3.gph" ,replace

    hist d365 if d365 <20 & cat ==4 , percent xlab(0/20) bin(20) ylab(0(20)60) ylabel(,angle(0)) title(Bronchospasm codes)
       gr play fig_hist_indiv.grec
       graph save Graph "C:\Users\8core\Documents\Bronchiolitis_ibuprofen\Maps\BMC revision\graph_cat4.gph",replace


  hist d365 if d365 <20 & cat_narrow ==1 , percent xlab(0/20) bin(20)  ylab(0(20)60) ylabel(,angle(0)) title(Bronchiolitis narrowly defined)
     gr play fig_hist_indiv.grec
     graph save Graph "C:\Users\8core\Documents\Bronchiolitis_ibuprofen\Maps\BMC revision\Graph_cat_narrow.gph" ,replace

  hist d365 if d365 <20 & cat_broad ==1 , percent xlab(0/20) bin(20) ylab(0(20)60) ylabel(,angle(0)) title(Bronchiolitis broadly defined)
   gr play fig_hist_indiv.grec
   graph save Graph "C:\Users\8core\Documents\Bronchiolitis_ibuprofen\Maps\BMC revision\graph_hist_4_broad.gph" ,replace



  #delimit ;

    grc1leg  Graph_cat_narrow.gph  graph_hist_4_broad.gph  graph_cat1.gph
        graph_cat2.gph graph_cat3.gph graph_cat4.gph

        ,col(2) title(Subsequent visits for wheezing at one year)
     ;

  #delimit cr
    gr play hist_fig_cleanup1.grec
    gr play hist_fig_cleanup2.grec


    graph save Graph "C:\Users\8core\Documents\Bronchiolitis_ibuprofen\Maps\BMC revision\Graph_all_cat.gph", replace
```
