# Supplementary material for: Which ICD-9-CM codes should be used for bronchiolitis research?
Source: BMC Med Res Methodol. 2018 Nov 22;18:149. doi: 10.1186/s12874-018-0589-4 (PMC6249877; doi:10.1186/s12874-018-0589-4)
Supplement: Supplementary file 5 — Stata_do_file_survival_graph. Stata do file. (PDF 26 kb) [file 12874_2018_589_MOESM5_ESM.pdf]

```
// creating overlaid survival curves

use "C:\Users\8core\Documents\Bronchiolitis_ibuprofen\Maps\BMC revision\surv_analysis_for_BMC_revision.dta"

gen set =0
save "C:\Users\8core\Documents\Bronchiolitis_ibuprofen\Maps\BMC revision\s1.dta" ,replace


clear
use "C:\Users\8core\Documents\Bronchiolitis_ibuprofen\Maps\BMC revision\surv_analysis_for_BMC_revision.dta"


gen set =1

save "C:\Users\8core\Documents\Bronchiolitis_ibuprofen\Maps\BMC revision\s2.dta" ,replace

clear

use s1.dta


/////////////////////////
///////////////////////////// Survival analysis set up

cap drop s_an6
cap drop s_an12
cap drop s_an24


bys id (cum_time_elapsed ): egen s_an24 = max(inception) if age[1] <=24
bys id (cum_time_elapsed ): egen s_an12 = max(inception) if age[1] <=12
bys id (cum_time_elapsed ): egen s_an6 = max(inception) if age[1] <=6

bys id (age ): replace s_an6 =0 if age[1] >6
bys id (age ): replace s_an12 =0 if age[1] >12
bys id (age ): replace s_an24 =0 if age[1] >24


lab var s_an6  "Include in survival analysis for <=6 mth group"
lab var s_an12 "Include in survival analysis for <=12 mth group"
lab var s_an24 "Include in survival analysis for <=24 mth group"


//////////////////////////////
//////////////////////////////

bys id (age ): replace cat  =cat[1]  if cat==.
bys id (age ): replace cat_narrow  =cat_narrow[1]  if cat_narrow==.

////////////////////
////creating two way graphs of survival


gen cat3 =1 if cat_narrow ==1 & set ==0
replace  cat3 =2 if cat_narrow ==0 & set ==1
tab cat3 ,mis
drop if cat3 ==.



 stset cum_time_elapsed, id(id7) failure(d365) exit(age==24)
sts graph ,adjust(b2season ) fail tmax(365) by(cat3)
gr play survival.grec
graph export "C:\Users\8core\Documents\Bronchiolitis_ibuprofen\Maps\BMC revision\Figure_survival_multiple.png", as(png) replace


stset cum_time_elapsed, id(id7) failure(d365==1) exit(age==24)
```

```
sts graph ,adjust(b2season ) fail tmax(365) by(cat3)
gr play survival.grec
 //manual edit title
graph save Graph "C:\Users\8core\Documents\Bronchiolitis_ibuprofen\Maps\BMC
revision\survival _first_visit.gph"


grc1leg "survival _first_visit.gph" Figure_survival_multiple.gph, xcomm ycomm col(1)

```
